# The Commercial Application of Insect Protein in Food Products: A Product Audit Based on Online Resources

**DOI:** 10.3390/foods13213509

**Published:** 2024-11-01

**Authors:** Lei Cong, David Dean, Chunguang Liu, Ke Wang, Yakun Hou

**Affiliations:** 1 Department of Agribusiness and Markets, Lincoln University, Lincoln 7647, New Zealand; david.dean@lincoln.ac.nz (D.D.); aidan.liu@lincoln.ac.nz (C.L.); 2 College of Food Science and Technology, Hebei Agricultural University, Baoding 071051, China; kewang_nz2018@outlook.com (K.W.); yakunhou86@hotmail.com (Y.H.)

**Keywords:** insect protein, alternative protein, product audit, protein-enhanced food, nutritional profiling, product nutritional audit

## Abstract

Insect protein has received considerable attention as an alternative to conventional animal proteins with its high nutritional contents and eco-friendly credentials. Exploring commercially available insect-protein-enhanced foods, this study aims to profile and compare such products in the ultra-processed category with products protein-enhanced with dairy (e.g., milk and whey) and plants (e.g., pea and rice). A global product audit was conducted drawing from English-language online retail portals to determine the product formats and statistically compare their nutritional contents with products fortified with non-insect proteins. The results show that four categories—flour/powder, pasta/noodle, starch-based snacks (e.g., chips, crackers, and cookies), and energy bars—are involved with food enhanced with insect protein. Flour/powder and pasta/noodles with insects demonstrated comparable protein contents to non-insect equivalents, highlighting insects’ potential as effective protein sources. However, insect protein’s performances in snacks and energy bars were less favourable, with significantly lower protein contents compared to products enhanced with non-insect sources. This may be attributed to the high fat content of insects, which may also contribute to undesirable flavours in complex foods, limiting their usage. The study highlights the need for industry innovation and scientific collaboration to overcome the challenges to widely applying insects as food ingredients, offering benefits for both the industry and consumers.

## 1. Introduction

A significant dilemma has emerged in light of the challenges faced in conventional animal protein production, such as land scarcity, overfishing, the looming threat of climate change [1], and the rapid increase in the global population, necessitating a staggering 70% increase in food production, and a two-fold surge in demand for livestock products by 2050 [2,3]. In response to these pressing concerns, alternative protein sources, including plants, edible insects, lab-grown organisms, and by-products of food production, have gained prominence in both the industry and academic community. These alternatives offer potential solutions to mitigate the adverse impacts of conventional animal protein production on a global scale [4], with edible insects emerging as a particularly noteworthy option.

Insects play a significant role in our world, with over 1 million species existing globally, 2000 of which serve as food and feed options [5,6]. They offer diverse edible forms, including eggs, larvae, pupae (chrysalis and puparia), and adults, although not all species are edible at every stage of development [6]. Across the world, trends that favour health benefits and environmental sustainability are driving the development of the edible insect industry [7]. Regulatory approvals for insect-based products have been obtained in various countries. For example, the following four insects have been approved for use as foods by the European Union: house cricket, yellow mealworm, migratory locust, and lesser mealworm [8]; in New Zealand and Australia, three edible insect types, super mealworm (*Zophobas morio*), mealworm beetle (*Tenebrio molitar*), and the house cricket (*Achaeta domestica*), have been reviewed and classified as non-traditional (not widely consumed) and non-novel, meaning they do not need to be formally assessed by the governmental authority to establish their food safety before being added to the food supply [9,10]. Globally, the edible insect industry is rapidly developing into a prosperous sector, with a global market size valued at USD 1.23 billion in 2023 and expected to reach around USD 9.14 billion by 2034 [11].

When considering nutritional profiles, in contrast to many plant-based protein sources, insects closely align with conventional animal products. Insects are rich in essential nutrients, including abundant protein contents ranging from 33% to 60% [12] and fat levels between 7% and 77% [13]. Moreover, they are packed with essential vitamins, fibre, and minerals [13], with impressive digestibility rates ranging from 78% to 98% [14]. Edible insects also offer a spectrum of additional benefits to human health, including enhanced immunity, gut regulation, and potential cancer prevention [15]. Compared with conventional meat protein, insects generally exhibit higher contents of crude protein, providing an optimal balance of the amino acids essential for human health, which meets the requirements set by the World Health Organization [16]. Specifically, insect protein is notably rich in lysine and methionine while relatively low in threonine and phenylalanine [13]. This nutrient composition positions insect protein as a promising alternative protein, offering not only nutritional benefits but also potential solutions to address global food security challenges.

From an environmental perspective, developing the insect industry could offset the environmental damage of conventional livestock products [6]. Insect cultivation generates reduced greenhouse gas emissions, demands less water and space, entails lower economic investment costs, and exhibits higher feed-conversion efficiency compared to conventional livestock farming [17]. Insects have a short growth cycle, which boosts food production efficiency and conserves resources. They occupy minimal space in ecosystems, unlike conventional livestock, reducing pressure on land and preserving ecosystem diversity [15]. Insects have low feed requirements and can consume various organic wastes, addressing waste issues. In contrast, conventional livestock farming demands extensive feed and land resources [12,18]. Insect farming, such as cricket and mealworm cultivation, uses minimal water, offering advantages over conventional animal husbandry [6]. This makes insect farming feasible in water-scarce areas like arid regions.

However, despite being a burgeoning industry with diverse applications [19], the integration of insects into diets faces several challenges in terms of factors influencing consumers’ low acceptance, e.g., food neophobia [14] and negative stereotypes towards insect consumption [20]. Moreover, plant-based proteins have consistently been found to be much more readily accepted than insect proteins, further complicating efforts to promote insects as a food ingredient [21]. Previous research has emphasised that a key psychological barrier to adopting insect protein in consumers’ diets is the visible presence of insects [22,23]. Since consumers are not yet ready to embrace the idea of consuming insects due to their unappealing appearance, the insect market is adopting innovative approaches in product development, incorporating insects into familiar dishes, essentially making them ‘invisible’ to consumers [24]. Such an example would be using cricket flour as a replacement for some of the wheat flour when producing cookies [13]. In the current study, products containing ultra-processed insect protein were chosen if they were enriched with insect-derived ingredients and the original insect shape was obscured. Hence, insect ingredients can be used not only to enhance the protein content of food products but also to retain the taste and familiarity of those products for consumers, which helps integrate insects into peoples’ daily diets.

In summary, edible insects present promising potential as an alternative protein source, especially for enhancing processed food products familiar to consumers. While numerous food products enhanced with insect proteins are available on the market, current studies have largely focused on biological features of and consumer attitudes towards insects, leaving a gap in the understanding of the product information of such, particularly regarding their applicable product formats and nutritional performance compared to traditional protein sources, such as dairy (e.g., milk and whey) and plant (e.g., pea and rice) enhancers. To fill this gap, this study comprehensively audited the commercial applications of processed food products enhanced with insect protein, based on a rich selection of online resources. Achieved by conducting thorough product reviews, the objective of this study was to explore the commercial applications of insect protein in the existing food market, addressing the following questions:

- What kinds of products enhanced with insect protein are currently available on the market?
- How does the nutritional performance of products enhanced with insect protein differ from those fortified by traditional protein enhancers?

The insights gained from this study can help with food industry innovation by highlighting opportunities for incorporating insect protein into product development, while also providing policymakers with the information needed to develop regulations in this field.

## 2. Materials and Methods

### 2.1. Data Collection

Following the guidelines in [25], the data collection for auditing human food products containing insect ingredients from online sales platforms was conducted in August 2023. Given the limited availability of food products enhanced with insect protein, conducting a physical product audit posed significant challenges, as such items are rarely found in supermarkets. The scarcity of these products also made it difficult to rely on specific online shopping platforms. To overcome this limitation, the Google Search engine was employed with English keyword combinations to locate and select products.

The initial search involved broad combinations of English keywords such as ‘insect’, ‘food’, and ‘products’, alongside more specific terms related to commonly used insect species (e.g., ‘cricket’, ‘mealworm’, and ‘locust’) to capture a diverse range of products. The Search was conducted methodically, with detailed browsing of the first 50 pages of Google Search results or until no new relevant products were identified. As the search progressed, additional keyword combinations, including terms such as ‘flour’, ‘powder’, ‘cookie’, and ‘energy bar’, were integrated based on earlier results to further expand the search scope. This broader search approach allowed us to locate and select products enhanced with insect protein across various sources, including brands’ official website and third-party sales platforms; ensuring a more comprehensive and inclusive product audit. To ensure the data’s reliability, product selection criteria were set, including:

- Food products for human consumption with ultra-processed insect protein as an ingredient;
- Consumer products;
- Prepacked products;
- Products available on the market, e.g., in-stock or available status;
- Products with reliable traceability information, e.g., manufacturer address and website;
- Products sold on reliable platforms, e.g., brands’ official website or third-party sales platforms;
- Products with clear nutritional information.

Products that did not meet the selection criteria were excluded. The information sources for the final selected products are available in the Supplementary Material 1. Product formats were summarised and grouped into several categories. Nutritional information on the selected products, such as protein, calories, fat, carbohydrates, sugars, fibre, cholesterol, etc., were collected and converted to comparable units—per 100 g.

Once the product format categories were established, an equal number of food products enhanced with traditional protein enhancers, e.g., plants and dairy, were found. These products are generally more accessible through many specific online shopping platforms than products enhanced with insects. Among the retail platforms, Amazon.com was selected as the source for sample collection due to its significant market presence and extensive range of offerings [26]. The platform’s comprehensive inventory and robust search functionality rendered it the most suitable option for the systematic identification of food products enhanced with traditional protein sources. For each of the product categories of insect foods, the most popular non-insect products were located, with the selection criteria mirroring that of the insect products. The same method was applied to gather nutritional information for each selected product.

Both datasets of products enhanced with insect and non-insect proteins were further checked by a second independent auditor to ensure data consistency and accuracy.

Detailed descriptions of the data collection steps are available in Supplementary Material 2.

### 2.2. Data Analysis

The products were organised into groups, based on protein sources and product categories, and then the nutrition information of the selected products was entered into a spreadsheet, encompassing protein, calories, total fat, saturated fat, carbohydrate, sugar, sodium, fibre, cholesterol, calcium, iron, and potassium. Not all of this nutritional information was available across all of the product types, so a maximum threshold of 20% missing data was set. The flour/powder products had the most detailed nutritional information, so more categories were available for analysis. SPSS 28 was used to first describe the means of the nutritional information across the groups of products [25]. Then, one-way ANOVAs were performed to establish differences across the groups. One-way ANOVAs are a commonly used test to compare mean differences across groups, but the technique requires that variables are normally distributed. Shapiro–Wilks tests indicated that the ingredient scores were not normally distributed, so the ingredient data were subjected to Box–Cox transformations, and the one-way ANOVAs were re-run using the transformed data. For the flour/powder products, additional post hoc tests were performed to establish significant differences among the groups. There are post hoc tests that assume homogeneity of variance and those that do not. The Levene’s tests indicated that homogeneity of variance was not present, and, thus, the Games–Howell post hoc test was deemed to be appropriate. In summary, a significant one-way ANOVA indicates that the ingredient significantly varies across the 2 or 3 groups in question. If two groups, this can confirm that the mean differences between the groups are significant. For 3-group ANOVAs, post hoc tests are required to determine whether differences between any 2 groups are significant.

## 3. Results

### 3.1. Formats of Products Enhanced with Insect Protein

The comprehensive search for products enhanced with insect protein that met the search criteria resulted in a wide variety but limited number of products. These products are available in various formats, including flour, powder, pasta, noodles, chips, crackers, cookies, and energy bars. Based on the ingredient complexity, four categories were utilised to group these product formats. These are flour/powder, pasta/noodles, starch-based snacks (chips, crackers, and cookies), and energy bars. The number of products under each category can be found in Table 1. In total, 71 products incorporating insect protein that met the search criteria were included in the audit. As illustrated in Table 1, information on an equivalent number of products with non-insect protein enhancers was collected and analysed. It is worth noting that products in the flour/powder category typically consisted of 100% of the main ingredient. Therefore, this category was further divided to allow for a comprehensive comparative assessment between insect protein and two traditional protein enhancers—plant and dairy.

### 3.2. Nutrition Comparison of Flour/Powder

Previous research has indicated that the most accepted form of edible insects is insect flour/powder, the transformation of which loses the original insect shape. This alteration plays a crucial role in reducing consumers’ fear and aversion to novelty [23]. Consequently, the insect flour/powder currently available on the market is primarily utilised as an ingredient, added to familiar foods to enhance protein content. It is essential to highlight that all insect flour products collected for this study exhibited a high insect content, often achieving 100% purity.

In addition, the traditional protein-fortified flours were composed mainly of plant-based protein ingredients, such as pea protein, brown rice protein, and rice protein, alongside dairy-based proteins, e.g., milk protein, whey protein, and protein isolate. A statistical descriptive analysis was conducted on the nutritional components of the flour/powder products enhanced with either insect or non-insect sources, and the average values are presented in Table 2.

**Figure 1:**
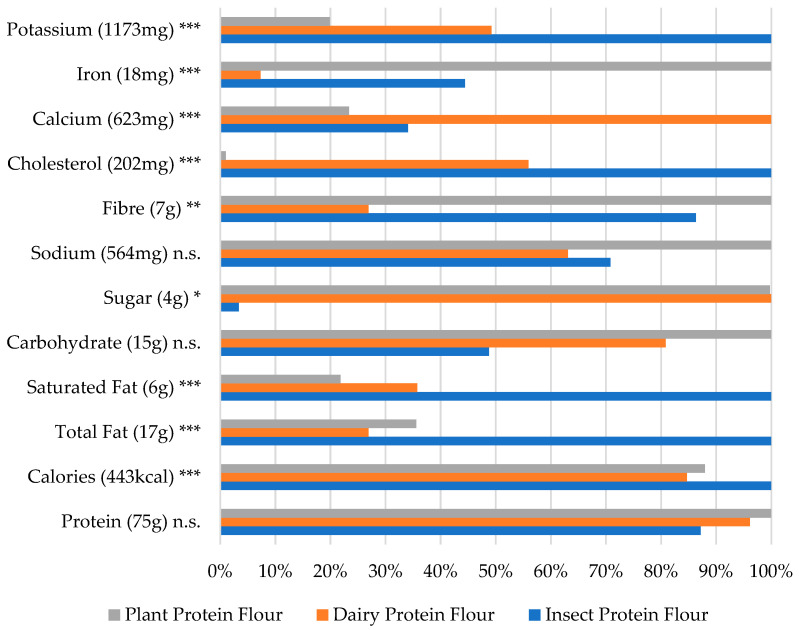
Nutritional values by protein source in the flour/powder category (% of largest value). The largest value (representing 100%) is indicated next to the ingredient name * *p* <0.05, ** *p* < 0.01, *** *p* < 0.001 and n.s. = not significant (based on the one-way ANOVA results).

To facilitate a more intuitive display and comparison of the variations among different product categories, this study opted for the use of a maximum value percentage format. In this method, the highest value within the same family of data was designated as 100%, serving as a reference point for comparing other data and generating a nutritional comparison chart across various categories. For example, plant-based powder is given a percentage of 100% for its protein content because it was higher (75.2464) than the dairy-based (72.337) or insect-based (65.6161) ones. Then, the other products are displayed as a percentage of the plant-based value resulting in percentage values for the dairy-based (72.337/75.2464 = 96.1%) and for the insect-based (65.6161/75.2464 = 87.2%) powders. The nutritional comparison of the flour/powder products enhanced with various protein sources is illustrated in Figure 1.

As shown in Figure 1, the protein contents among the three types of products exhibited remarkable similarity, with no significant differences. This suggests that the functionality of insect protein is on par with other fortified proteins. Moreover, the insect product stands out for its low sugar (*p* < 0.05) content, presenting a noticeable advantage, particularly aligning with the growing demands for health-conscious and low-sugar foods. In comparison to the other two product types, the insect-based flour/powder contained higher levels of the micronutrient potassium (*p* < 0.001). This could potentially serve as a unique feature that appeals to consumers who are specifically seeking to supplement potassium. In summary, insect flour/powder proves to be ideal for developing products that demand a combination of high protein, high potassium, and low sugar.

However, the fat content of edible insects tends to be high, exemplified by crickets, for which fat constitutes the largest nutritional component, at approximately 21.8% [27]. Figure 1 illustrates the elevated levels of saturated fat (*p* < 0.001), total fat (*p* < 0.001), and cholesterol (*p* < 0.001) in the insect products, which were significantly higher than for products enhanced with plant and dairy. Consequently, the high composition of fat-related components contributed to the high-calorie content (*p* < 0.001). Considering the prevailing trend towards low-fat dietary choices today, this elevated fat content may pose a potential disadvantage for insect powder.

### 3.3. Nutrition Comparison of Products with Complex Ingredients

**Figure 2:**
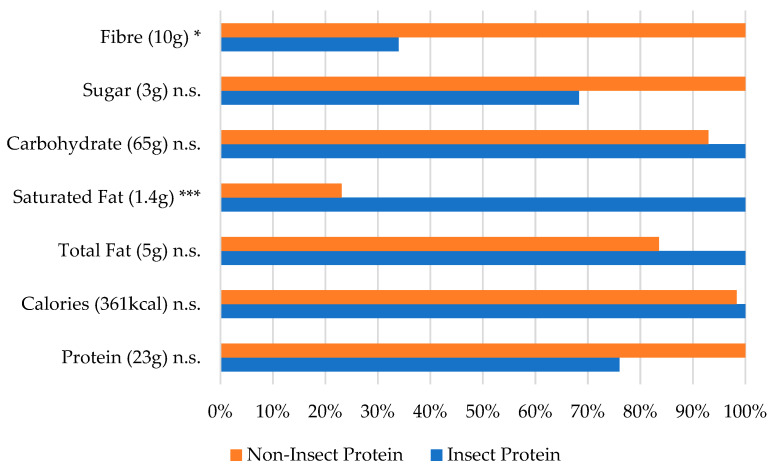
Nutritional values by protein source for the noodle/pasta category (% of largest value). The largest value (representing 100%) is indicated next to the ingredient name. * *p* < 0.05, *** *p* < 0.001, and n.s. = not significant (based on the one-way ANOVA results with Box–Cox transformation).

**Figure 3:**
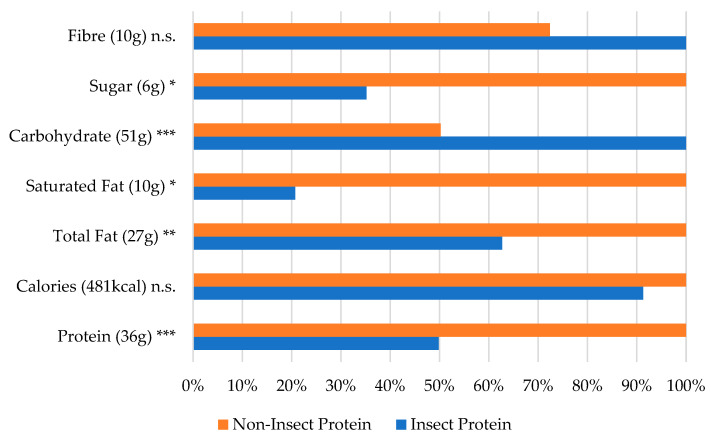
Nutritional values by protein source for the starch-based snack category (% of largest value). The largest value (representing 100%) is indicated next to the ingredient name. * *p* < 0.05, ** *p* < 0.01, and *** *p* < 0.001 (based on the one-way ANOVA results with Box–Cox transformation).

**Figure 4:**
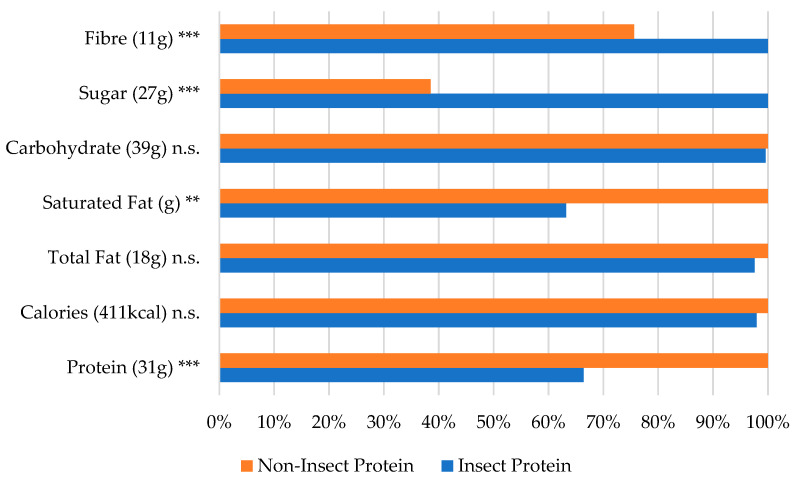
Nutrition values by protein source in the energy bar category (% of largest value). The largest value (representing 100%) is indicated next to the ingredient name, ** *p* < 0.01, *** *p* < 0.001, and n.s. = not significant (based on the one-way ANOVA results with Box–Cox transformation).

Insect flour/powder is commonly incorporated as a partial ingredient in insect-based products directly consumable by humans [23]. These foods contain complex ingredients, including products in the categories of pasta/noodle and starch-based snacks. Many of them exhibit diverse flavour characteristics, for example, insect-protein potato chips of various flavours and chocolate/fruit energy bars. The audit’s results and statistical findings are summarised in Table 3 and Figure 2, Figure 3 and Figure 4. All products involving non-insect-protein enhancers are considered as a single group under each product category.

In Figure 2, describing the maximum value percentage of noodles/pasta, significant differences were observed but only in the fibre and saturated-fat levels across insect- and non-insect-enhanced products. However, the nutritional composition of other elements, such as protein, remained similar, with no significant differences observed. This finding suggests that noodles/pasta enriched with different protein sources present comparable nutritional profiles, except for the low-fibre and elevated saturated-fat contents in insect-enhanced products.

Focusing on the starch-based snacks, Figure 3 illustrates a significantly lower protein content (*p* < 0.001) in insect-enhanced products compared with non-insect foods, despite the advantages of lower saturated-fat (*p* < 0.05) and total-fat (*p* < 0.01) contents in the insect products. A comparison across energy bars, shown in Figure 4, reveals similar results in terms of the protein (*p* < 0.001) and saturated-fat (*p* < 0.01) contents in starch-based snacks.

In summary, the analysis of the three categories of complex ingredient products reveals that only the pasta/noodles enhanced with insect protein exhibited a protein content comparable to non-insect products. However, the results for starch-based snacks and energy bars are less satisfactory. While insects can serve as protein fortifiers in processed foods, the nutritional contents of the current insect-enhanced products on the market do not exceed those of other non-insect enhancers. From a nutritional performance perspective, products with simpler ingredients, such as pasta/noodles, may outperform complex foods such as snacks and energy bars.

## 4. Discussion

The development and utilisation of insect protein resources have entered an active stage, with a growing number of commercial and retail products, progressing toward industrialisation and scale [1]. Despite the numerous advantages of insect protein, with clear evidence from the laboratory data, including sustainability and protein content, its commercial application in food remains a considerable challenge. For example, although this study employed online resources to collect as many products as possible in this field, only the following four general product categories were identified: flour/powder, pasta/noodles, starch-based snacks, and energy bars. The number of products within each category was also relatively limited. This reflects the early stage of the insect protein market. Moreover, the dynamic nature of this market is demonstrated by the frequent occurrence of product discontinuation and iteration. Many products are either withdrawn from retail shelves or modified in response to changing consumer preferences and industry trends. Consequently, while this study provides valuable insights into this emerging market, the findings, despite offering a comprehensive nutritional analysis of the 71 products identified, should be viewed as preliminary due to the limited sample size.

On the other hand, because of the comprehensive product audit, the findings of the current study reveal that insects can serve as a competitive protein enhancer compared with traditional sources such as plant and dairy proteins. This is especially reflected in products with simple ingredient formats, such as flour/powder and pasta/noodles. Additionally, as a food ingredient, insect protein also offers advantages such as lower sugar/carbohydrate content and higher potassium levels, potentially benefiting specific consumer groups seeking a low-carbohydrate and/or high-potassium protein enhancer. However, insect protein also presents the characteristics of high fat and cholesterol contents, which may not align with contemporary trends in healthy eating. In some complex food products, such as starch-based snacks and energy bars, the nutritional advantages of insect protein may not be well demonstrated. The reason for this might be due to the crucial role fat plays in the transmission of flavour molecules, thus limiting the amount of insect protein that can be added to processed foods before negatively impacting the desired taste [28]. Our research findings indicate that the adaptability of insect proteins varies across different product forms. Noodle/pasta products appear to be more suitable as carriers for insect proteins, whereas insect proteins exhibit relatively poor performance in cookies, crackers, potato chips, and energy bars. This may be caused by such products having higher flavour requirements, thereby limiting the amount of insect protein that can be added. According to previous studies, the proportion of crickets added to insect-enhanced products is typically limited to around 10% to maintain a favourable balance [27,29]. In contrast, plant-based and dairy-based proteins do not face the same taste-masking challenges, allowing manufacturers to increase the concentration of protein enhancers, potentially explaining the higher protein levels in non-insect-enhanced snacks and energy bars.

Based on previous research, there are numerous factors influencing consumer acceptance of insect protein. For instance, food neophobia [14], which is characterised by a reluctance to try new foods and is influenced by variables like age, gender, and education level, contributes to low acceptance rates [27]. Negative perceptions and stereotypes surrounding insect consumption, particularly in Western societies, further hinder widespread adoption [20]. Additionally, demographic characteristics, such as age, knowledge, and education level, play crucial roles in shaping acceptance, with younger demographics and those with higher education levels displaying greater openness [30,31]. Social factors, including family and peer influence, also significantly impact consumer choices [20]. Product attributes, such as sensory characteristics, nutritional value, packaging, and pricing, further influence acceptance [14,24]. Lastly, safety concerns could be an obstacle to influencing consumers’ frequency of consumption [32].

However, efforts to alleviate these through education, awareness, and familiarisation with insect foods, especially concerning the high protein content and sustainable features, can help shift consumer perceptions [33]. The results of this, based on evidence from existing market applications of insect protein, can assist consumers and manufacturers in understanding the product formats and nutritional performance of insect protein. Taste is one of the critical influencers of consumer acceptance [22], and the current study suggests that products or technologies that somehow overcome the unwanted taste of insect protein would be in high demand. For example, the development of low-fat insect protein would be advantageous, serving to both minimise unwanted flavour and reduce calorie content.

Although insect protein is recognised as an excellent alternative source, studies related to its application in processed human foods remain limited. To foster growth in the insect protein industry, it is encouraged to promote its incorporation into a broader range of product formats, thereby expanding its potential applications to improve taste and broaden consumer acceptance. Moreover, it is crucial to consider the diverse dietary preferences and needs of different consumer groups when promoting insect protein products [2]. For instance, in Western markets, where sustainability is highly valued, emphasising the sustainable attributes of insect-protein products is essential to cater to this demand [34]. The low-carbohydrate and high-potassium features of insect protein could be valuable to certain groups of consumers who demand it. Furthermore, a thorough analysis of product pricing, including factors such as technological advancements, production costs, and logistics, is essential for devising targeted marketing strategies and product positioning strategies to enhance the global appeal of insect proteins [35].

## 5. Practical Implications

The study highlights that insect protein has begun to appear in complex products, such as chips, crackers, cookies, and energy bars, and the variety of these products remains limited. The inclusion of insect protein is often minimal due to challenges related to the high-fat content and associated flavour issues. This suggests a crucial need for the food industry to explore and develop a broader range of product types that can effectively incorporate insect protein. To address these challenges, collaboration between food scientists and industry practitioners is essential. Technological advancements aimed at reducing fat and improving flavour profiles are vital for enhancing the integration of insect protein into diverse products. Additionally, the findings from this study provide valuable insights that can assist policymakers in formulating evidence-based regulations and standards, which can further support the expansion of insect protein applications in the food market, fostering industry innovation and aiding in the development of new products that meet consumer demands and regulatory requirements.

## 6. Future Research and Limitations

While the study provides valuable insights regarding commercialised food products enhanced with insect protein, several limitations exist. First, as a preliminary study, the sample size of 71 products, despite being the most comprehensive collection can be conducted, remains statistically limited. The frequent introduction of new products, as well as product modifications and discontinuations, suggests that the landscape may change significantly over a short period of time. Therefore, future research should aim to capture a broader and more representative sample as the market matures. Moreover, since the study utilised English-language online sources to maximise product variety, this approach may not fully capture insect-based products promoted in other languages. Future research should include a broader range of online sources, navigating various languages to obtain a more comprehensive view of the products in regional markets. Additionally, the study focused on products explicitly claimed by producers to be protein-enhanced, excluding other claims such as high-fibre and low-sugar contents. Future investigations should explore these additional product categories to better understand the diverse applications of insects as food ingredients. Another aspect for future studies to explore is the use of multiprotein enhancers, such as combining insect protein with dairy protein in a single food product. Investigating how these combinations affect the overall nutritional value and market appeal could provide valuable insights into their potential benefits and limitations. Moreover, the current research was limited to human food products; therefore, expanding the scope to include insect protein in pet foods and other non-human applications could provide further insights into its potential uses and market opportunities.

## 7. Conclusions

While numerous studies have confirmed that insects boast high protein content, positioning them as an excellent alternative protein substitute or enhancer poses some challenges. This study audited the available formats of products enhanced with insect protein and documented the nutrient contents of food products containing insect proteins compared to traditional sources of protein. The results show that, based on available online resources, ultra-processed insect protein has been used to enhance the protein levels of the following four types of ultra-processed food products: flour/powder, pasta/noodles, starch-based snacks (chips, crackers, and cookies), and energy bars.

Within these categories, flour/powder typically comprise purely insect-derived ingredients and are used as an ingredient in the production of complex food products. Upon careful examination, both flour/powder and pasta/noodles have protein contents comparable to traditional protein enhancers. This underscores the potential of insects as robust protein fortifiers. Additionally, when compared to traditional plant and dairy flour/powder, insect protein features low sugar/carbohydrate contents and high potassium levels. However, the efficacy of insect protein within complex food products appears to be limited. This could be attributed to the fact that certain complex ingredient foods have high flavour requirements, and since insects contain high levels of fat-related components, they may present undesirable flavours if used in high concentrations. Consequently, the amount of insect protein that can be added is restricted. Hence, it follows that food items with less demanding flavour requirements could potentially serve as more conducive platforms for integrating higher levels of insect protein.

As an excellent alternative protein source, the high-fat content of insect protein not only limits its usage in complex foods but also contradicts modern trends in healthy diets. Thus, the insect protein sector must focus on innovating methods to decrease the fat content in insect-based products. Furthermore, given the unique nutritional features of insect protein, manufacturers are suggested to develop a range of insect products tailored to meet the diverse nutritional requirements and preferences of consumers. Future research endeavours should focus on enhancing product taste, refining marketing strategies, and promoting sustainable development to facilitate the wider adoption of insect protein in the food industry.

In summary, our research contributes to bridging existing knowledge gaps and providing a foundation for future research through a comprehensive audit of insect protein-enhanced food products, offering potential benefits across academic, manufacturing, marketing, and policy-making domains.

## Figures and Tables

**Table 1:** Category and quantity of audited products.

Category	Insect Product	Non-Insect Product	
Flour/powder	19	Plant	Dairy
		19	19
Pasta and noodle	11	11	
Starch-based snack	15	15	
Energy bar	26	26	

**Table 2:** Mean contents of nutrients per 100 g of flour/powder.

Nutritional Information	Protein Source Means			One-Way ANOVAs (Box–Cox)	
	Insect	Dairy	Plant	F Statistic	*p*-Value
Protein (g)	65.6161	72.3317	75.2464	1.887	0.161
Sugar (g)	0.1250	3.7157_I_	3.7062	3.568	0.035
Carbohydrate (g)	7.0874	11.7451	14.5292	1.189	0.312
Saturated Fat (g)	6.0713_DP_	2.1689	1.3248	23.437	<0.001
Total Fat (g)	17.1969_DP_	4.6257	6.1136	28.904	<0.001
Cholesterol (mg)	202.3520_P_	113.1487_P_	0.000	17.458	<0.001
Calories (kcal)	443.5045_DP_	375.6421	390.0334	24.656	<0.001
Sodium (mg)	399.8846	356.2395	564.6057	1.981	0.148
Calcium (mg)	212.2910	623.3526_IP_	145.6408	14.168	<0.001
Iron (mg)	7.9798_D_	1.3194	17.9721_ID_	13.024	<0.001
Potassium (mg)	1173.7776_P_	577.7199_P_	233.4046	13.570	<0.001
Fibre (g)	6.2133	1.9365	7.1970	5.562	0.006

I = greater than insect; D = greater than dairy; P = greater than plant (Games–Howell post hoc test, *p* < 0.05).

**Table 3:** Mean nutritional contents of the complex ingredients of products enhanced with insect and non-insect proteins.

	Protein Source Means		One-Way ANOVAs (Box–Cox)	
	Insect	Non-Insect	F Statistic	*p*-Value
Noodle/pasta				
Protein (g)	17.5956	23.1429	2.054	0.167
Sugar (g)	2.2355	3.2739	0.878	0.360
Carbohydrate (g)	64.5639	60.0125	0.805	0.380
Saturated Fat (g)	1.441	0.333	32.756	<0.001
Total Fat (g)	5.1959	4.3393	0.242	0.628
Calories (kcal)	360.5471	354.6091	0.083	0.776
Fibre (g)	3.4955	10.2918	5.872	0.025
Snack				
Protein (g)	17.8571	35.8417	18.827	<0.001
Sugar (g)	2.0448	5.809	6.826	0.014
Carbohydrate (g)	51.3457	25.7957	22.808	<0.001
Saturated Fat (g)	2.0038	9.663	6.959	0.013
Total Fat (g)	17.3152	27.6192	10.270	0.003
Calories (kcal)	439.3238	481.3504	3.392	0.076
Fibre (g)	9.6156	6.9594	1.497	0.231
Energy bar				
Protein (g)	20.7385	31.242	35.440	<0.001
Sugar (g)	27.2538	10.4964	30.774	<0.001
Carbohydrate (g)	38.3731	38.5351	0.004	0.951
Saturated Fat (g)	5.1654	8.1748	8.920	0.004
Total Fat (g)	17.7077	18.1482	0.058	0.810
Calories (kcal)	402.2846	410.6892	0.309	0.581
Fibre (g)	11.3308	8.5658	22.513	<0.001

## Data Availability

The original contributions presented in the study are included in the article, further inquiries can be directed to the corresponding author.

## Supplementary Materials

The following supporting information can be downloaded at: https://www.mdpi.com/article/10.3390/foods13213509/s1.
