# Supplementary material for: The Commercial Application of Insect Protein in Food Products: A Product Audit Based on Online Resources
Source: Foods. 2024 Nov 1;13(21):3509. doi: 10.3390/foods13213509 (PMC11545340; doi:10.3390/foods13213509)
Supplement: Supplementary file 1 [file foods-13-03509-s001.zip › foods-3205999-supplementary.pdf]

Supplementary Material 1

Format	Brand	Product	Information source
Flour/powder	Cricket flours	Pure powder, 100g	https://www.cricketflours.com/product/cricket-flour-cricket-powder/
Flour/powder	Thanland unique	Cricket powder (acheta domesticus), 100g	https://www.thailandunique.com/insect-bug-flour-powder/premium-cricket-flour-powder
Flour/powder	Thanland unique	Cricket powder (gryllus bimaculatus), 100g	https://www.thailandunique.com/insect-bug-flour-powder/cricket-flour-protein-powder#:~:text=Gryllus%20Bimaculatus%20crickets%20are%20raised,or%20flavours%20have%20been%20added.
Flour/powder	Thanland unique	Silkworm pupae powder (bombyx mori), 11g	https://www.bizarrefood.com/chocolate-covered-silkworms
Flour/powder	Hoppa	Cricket baking flour, 250g	https://www.hoppafoods.com/product/cricket-baking-flour/
Flour/powder	Exo	Pure cricket protein powder, 454g	https://www.amazon.com/dp/B07VXCN5LL/?tag=nanowerk-20
Flour/powder	Exo	Acheta (cricket) powder, 113g	https://exoprotein.com/collections/all-products/products/cricket-powder-powder
Flour/powder	Small giant	Cricket flour, 200g	https://eatsmallgiants.com/product/cricket-flour/
Flour/powder	Small giant	Buffalo worm flour, 200g	https://eatsmallgiants.com/product/buffalo-worm-flour/
Flour/powder	Eat crawlers	Cricket flour, 100g	https://eatcrawlers.co.nz/collections/cricket-flour/products/cricket-flour-100g
Flour/powder	Essento	Cricket flour, 50g	https://essento.ch/en/product/cricket-flour/
Flour/powder	Ento	Ento 100% pure cricket powder, 100g	https://www.ento.my/collections/roasted-crickets/products/protein-powder
Flour/powder	Mightycricket	Pure cricket flour (variable sizes), 14.6g	https://mightycricket.com/product/cricket-flour/
Flour/powder	Chapul cricket	Chapul cricket flour, 1lb	https://chapul.com/products/chapul-cricket-flour-protein
Flour/powder	Bug farm foods	Insect powder – cricket, 95g	https://www.thebugfarm.co.uk/shop/food/bug-farm-foods-insect-powder-cricket/?
Flour/powder	Chirps	Chirps cricket powder, 11 lbs	https://eatchirps.com/products/wholesale-cricket-protein-powder-11-lbs-shopify-product-page?_pos=1&_sid=b00415e1e&_ss=r
Flour/powder	Entomo	Cricket powder, 454g	https://ca.entomofarms.com/products/cricket-powder
Flour/powder	Circle harvest	Cricket protein powder, 200g	https://circleharvest.com.au/products/cricket-protein-powder-1-healthy-alternative
Flour/powder	Mighty cricket	Mighty cricket flour, 14.6g	https://mightycricket.com/product/cricket-flour/
Starch-based snack	Chirps	Cricket chips (bbq), 140g	https://chirps-chips-test.myshopify.com/products/chirps-variety-pack-three-5-oz-bags
Starch-based snack	Chirps	Cricket chips (cheddar), 140g	https://chirps-chips-test.myshopify.com/products/chirps-variety-pack-three-5-oz-bags
Starch-based snack	Chirps	Cricket chips (sea salt), 140g	https://chirps-chips-test.myshopify.com/products/chirps-variety-pack-three-5-oz-bags
Starch-based snack	Circle harvest	Cricket corn chips (bbq), 50g	https://circleharvest.com.au/collections/gluten-free-insect-protein/products/cricket-corn-chips-protein-snack
Starch-based snack	Circle harvest	Cricket corn chips (saltbush & rosemary), 50g	https://circleharvest.com.au/collections/gluten-free-insect-protein/products/cricket-corn-chips-protein-snack
Starch-based snack	Circle harvest	Cricket corn chips (lightly salted), 50g	https://circleharvest.com.au/collections/gluten-free-insect-protein/products/cricket-corn-chips-protein-snack
Starch-based snack	Sens	Pea cricket protein chips (poppy seeds & sea salt), 80g	https://www.caspersclimbingshop.com/nutrition/3880-sensproteinpeachipswithcricketflourandpoppyseeds.html
Starch-based snack	Sens	Protein chips (garlic & herbs), 80g	https://www.caspersclimbingshop.com/nutrition/7130-sens---cricket-protein-chips---garlic--herbs---80-g.html
Starch-based snack	Sens	Protein chips (hot pepper), 80g	https://www.caspersclimbingshop.com/nutrition/7131-sens---cricket-protein-chips---hot-paprika---80-g.html
Starch-based snack	Grub	Cricket corn chips, 75g	https://grubprotein.com.au/products/cricket-corn-chips
Starch-based snack	Small giants	Cracker bites (rosemary & thyme), 80g	https://www.jiminis.com/shop/lang/en/crackers-bites/299-cracker-bites-rosemary-thyme-5060633990210.html
Starch-based snack	Small giants	Cracker bites (turmeric & smoked paprika), 80g	https://www.jiminis.com/shop/lang/en/crackers-bites/305-crackers-turmeric-smoked-paprika-5060633990227.html?srsltid=AfmBOop4bseHeDUbNsseV40-5jhbTnMG4jP10pwx9DUhAOhRasmpRPJu
Starch-based snack	Fucibo	Extra ordinary chipst, 25g	https://21bites.com/products/fucibo-pizza-chips-with-insect-powder
Starch-based snack	Pinaks	Insektencracker rosmarin, 35g	https://www.startnext.com/pinaks/ds/d/insekten-cracker-i332232.html
Starch-based snack	Small giants	Cracker bites (tomato & oregano), 40g	https://21bites.com/products/small-giants-red-tomato-oregan-cricket-crackers
Pasta and noodle	Bugsolutely	Cricket pasta, 350g	https://www.bugsolutely.com/company/
Pasta and noodle	Eat crawlers tomo	Tomorrow foods protein pasta, 250g	https://eatcrawlers.co.nz/products/protein-pasta-powered-by-crickets-penne-250g?srsltid=AfmBOoq68ivDoydU27b_upoeauNlMpdG9CwaujY0mPDzuEBfVHPKdXq6
Pasta and noodle	Sens	Semolina penne (with gluten), 200g	https://eatsens.com/products/gluten-freecricket-protein-pasta
Pasta and noodle	Hoppa	Cricket pasta (penne), 250g	https://sites.google.com/view/hoppafoods-australia/
Pasta and noodle	Grub	Cricket pasta, 350g	https://grubprotein.com.au/products/cricket-pasta
Pasta and noodle	Jimini's	Fusilli protein pasta, 250g	https://www.jiminis.com/shop/lang/en/protein-pasta/307-pates-3760274101724.html
Pasta and noodle	Jimini's	Fusilli basil and spinach, 250g	https://www.jiminis.com/shop/lang/en/protein-pasta/307-pates-3760274101724.html
Pasta and noodle	Jimini's	Fusilli turmeric with insect powder, 250g	https://21bites.com/collections/new/products/jiminis-pasta-turmeric-fusilli
Pasta and noodle	Nutribug	Cricket protein pasta (concept product), 500g	https://nutribug.com/product/cricket-protein-pasta/
Pasta and noodle	Entomos	Acheta pasta, 200g	https://gourmetbugs.ch/fr/product/pates-proteinees-aux-grillons-200g-entomos--entomos1005?cat=
Pasta and noodle	Keero	Noodles cricket protein, 410g	https://theadcc.ca/archive/keero-cricket-protein-noodles_2022_silver_graphic-design
Energy bar	Essento	Protein bar (raw cacao), 35g	https://essento.food/en/products/raw-cacao-protein-bar?srsltid=AfmBOooIJha-8L64H6vOj6WxBXHxRA9ZwkJWrBU8dWPJ0-CRRl3P-2uG
Energy bar	Essento	Protein bar (berry almond), 35g	https://essento.food/en/products/berry-almond-protein-bar?srsltid=AfmBOooVuUi1Rre41o0QRBTCty32AM9UiY3rNrV9n28fBr6o_8qyKC9y
Energy bar	Jimini's	Apricot & almonds protein bar, 40g	https://www.jiminis.com/shop/lang/en/protein-bars/282-apricot-almond-protein-bar.html
Energy bar	Jimini's	Fig & chocolate bar,40g	https://www.jiminis.com/shop/lang/en/protein-bars/279-fig-and-chocolate-protein-bars.html
Energy bar	Jimini's	Banana and chocolate bar, 40g	https://www.jiminis.com/shop/lang/en/protein-bars/281-banana-chocolate-protein-bar.html
Energy bar	Jimini's	Apple and caramel bar, 40g	https://www.jiminis.com/shop/lang/en/protein-bars/280-insect-protein-bars-apple-and-caramel.html
Energy bar	Green bite	Raw power cricket bar, 50g	https://www.greenbite.ee/shop/superfood-bars/cricket-bars
Energy bar	Green bite	Bug challenge cricket bar, 50g	https://www.greenbite.ee/shop/superfood-bars/bug-challenge-cricket-bar-1
Energy bar	Sens	Cricket protein bar (dark chocolate-caramel & peanut), 60g	https://www.caspersclimbingshop.com/nutrition/7033-sens---cricket-protein-bar-in-dark-chocolate---chocolate-brownie.html
Energy bar	Sens	Cricket protein bar (chocolate brownie), 60g	https://www.amazon.de/-/en/Protein-Cricket-Fitness-Athletes-Brownie/dp/B0CGBZ3FC4
Energy bar	Sens	Cricket protein bar (choco & banana), 60g	https://eatsens.com/search?q=bar&options%5Bprefix%5D=last&type=product%2Carticle%2Cpage
Energy bar	Sens	Cricket protein bars (peanut butter & cinnamon),60g	https://eatsens.com/search?q=bar&options%5Bprefix%5D=last&type=product%2Carticle%2Cpage
Energy bar	Sens	Cricket protein bar (bitter cocoa &sesame), 60g	https://eatsens.com/search?q=bar&options%5Bprefix%5D=last&type=product%2Carticle%2Cpage
Energy bar	Sens	Pleasure cricket protein bars-dark chocolate, 60g	https://eatsens.com/search?q=bar&options%5Bprefix%5D=last&type=product%2Carticle%2Cpage
Energy bar	Sens	Cricket protein bar (pineapple & coconut bar), 60g	https://eatsens.com/search?q=bar&options%5Bprefix%5D=last&type=product%2Carticle%2Cpage
Energy bar	Sens	Cricket protein bar (dark chocolate &orange), 60g	https://eatsens.com/search?q=bar&options%5Bprefix%5D=last&type=product%2Carticle%2Cpage
Energy bar	Exo	Peanut butter chocolate chip protein bars, 50g	https://exoprotein.com/collections/featured/products/peanut-butter-chocolate-protein-bar
Energy bar	Exo	Cookie dough protein bars, 50g	https://exoprotein.com/collections/high-protein-bars/products/chocolate-chip-cookie-dough-protein-bars
Energy bar	Heyplanet	Cocoa orange protein bar, 45g	https://organicbeautysupply.eu/shop-eng/907-snacks/1353-hey-planet---insect-protein-bar-with-cocoa-orange/
Energy bar	Heyplanet	Apple cinnamon protein bar, 45g	https://www.hey-planet.com/products/protein-bar-cocoa-orange
Energy bar	Heyplanet	Salty peanut protein bar, 45g	https://www.hey-planet.com/products/protein-bar-cocoa-orange
Energy bar	Hoppa	Cricket energy bars (peanut butta nutta), 50g	https://www.hoppafoods.com/product/peanut_butter_and_oats_cricket-protein-energy-bar/
Energy bar	Hoppa	Cricket energy bars (caribbean crunch), 50g	https://www.hoppafoods.com/product/coconut_and_pumpkinseeds_cricket_protein_energy_bar/
Energy bar	Hoppa	Cricket energy bars (cinnamon slice), 50g	https://www.hoppafoods.com/product/cinnamon-and_chiaseeds-cricket-protein-energy-bars/
Energy bar	Hoppa	Cricket energy bars (whole lotta chocca), 50g	https://www.hoppafoods.com/product/cacao_and_cashewnut_cricket_protein_energy_bar/
Energy bar	Getsharp	Energy bar with mealworm powder, 50g	https://www.bienmanger.com/2F31467_Energy_Bar_With_Fruits_Chocolat_And_Insect_Powder.html

Data Collection Steps

1. **Define Initial Search Terms**:
   Search terms consist of specific keywords related to common insect species and general keywords linked to the research objectives.
   - The list of specific keywords: ‘cricket’, ‘mealworm’, and ‘locust’.
   - The list of general keywords: ‘insect’, ‘food’, and ‘products’.
2. **Conduct Google Search**:
   Various search queries are generated by combining specific and general keywords. Results from Google are systematically reviewed, browsing up to the first 50 pages. If no new products are identified within this range, the search is deemed exhaustive for that combination.
3. **Expand with Additional Keywords**:
   As the search progresses, additional keywords are incorporated to capture a wider array of relevant products.
   - The list of additional keywords: ‘flour’, ‘powder’, ‘cookie’, ‘snack’, ‘chips’, ‘crackers’, ‘pasta’, ‘noodle’ and ‘energy bar’.
4. **Select Insect Products**:
   Identified products are further selected according to predefined criteria:
   - Food products for human consumption with ultra-processed insect protein as an ingredient;
   - Consumer products;
   - Prepacked products;
   - Products available in the market, e.g. on-stock or available status;
   - Products with reliable traceability information, e.g. manufacturer address and website;
   - Products sold on reliable platforms, e.g. brands’ official website or third-party sales platforms;
   - Products with clear nutrition information.
5. **Collect Nutritional Data for Insect Protein Products**:
   All available nutritional information available in either the website or package of the products is collected for each product and standardised to per 100 grams.
6. **Categorise Insect Products**:
   Based on ingredient complexity, four format categories were utilised to group product enhanced by insect-protein. These are flour/powder, pasta/noodles, starch-based snacks (chips, crackers, and cookies), and energy bars.
7. **Select Traditional Protein Products**:
   Traditional protein-enhanced products were identified using Amazon’s embedded

search engine, given its dominant market presence and extensive product variety. To match each group of insect protein products, search terms were structured by combining ‘protein-enhanced’ with the specific product format, such as ‘protein-enhanced flour/powder’. The search results were then ranked by sales, and an equivalent number of products were selected to correspond with the insect protein categories. Since the flour/powder category typically consists of 100% of the main ingredient, it provides more comprehensive nutritional data for analysis and discussion. Therefore, when selecting the highest-selling products, an equal number of plant-based and dairy-based flour/powder were chosen for further comparison.

- The list of search terms: ‘protein-enhanced flour/powder’, ‘protein-enhanced pasta/noodle’, ‘protein-enhanced snack’ and ‘protein-enhanced energy bar’

8. **Collect Nutritional Data for Traditional Protein Products**:
   Nutritional data for selected traditional protein-enhanced products is collected following the same criteria used for insect-based products.

9. **Data Validation**:
   Both datasets, products enhanced by insect and traditional proteins, are independently reviewed by a second author to verify consistency and accuracy.
